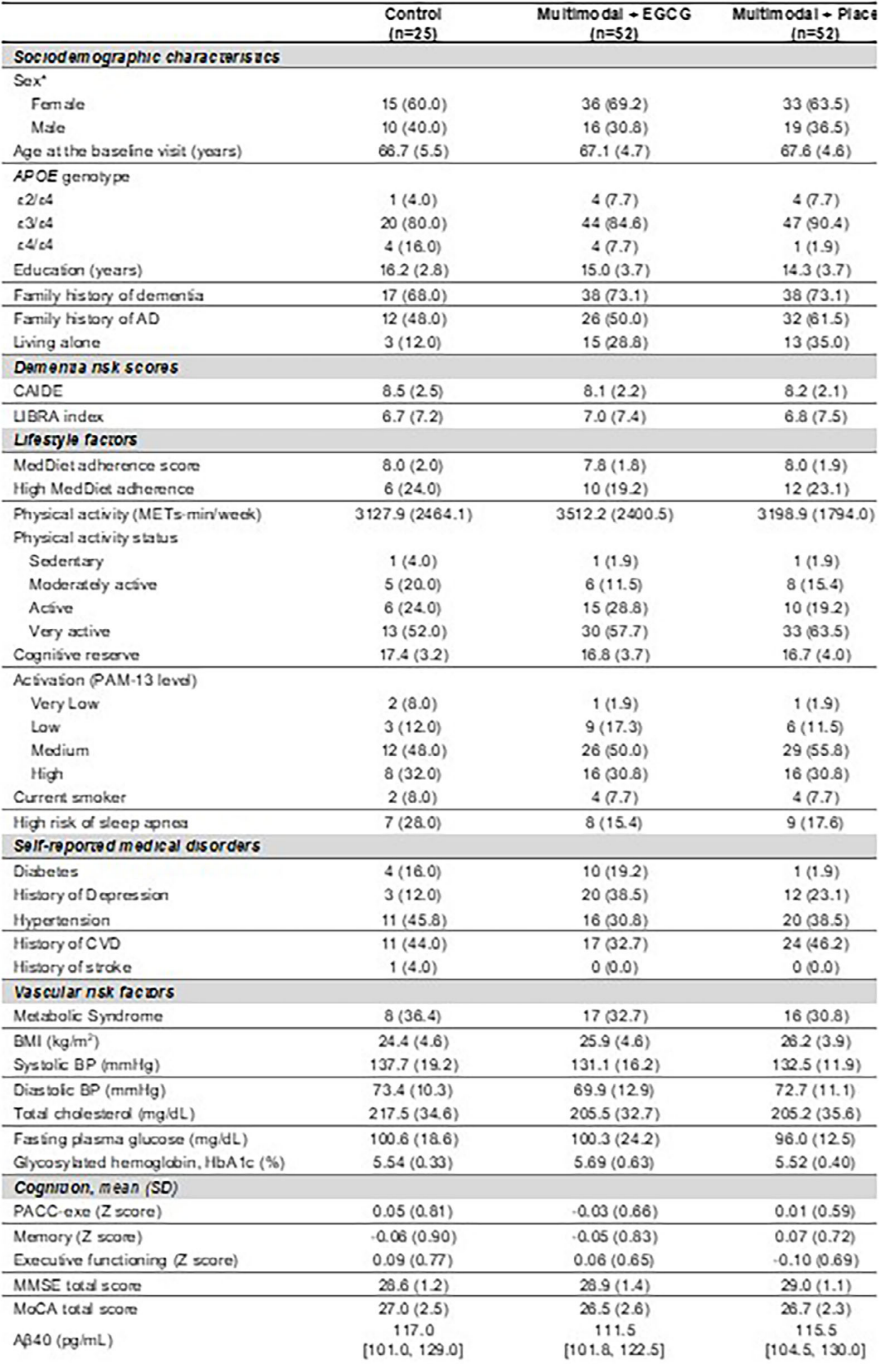# PENSA study, results of a 12‐month personalized intensive multimodal lifestyle intervention complemented with epigallocatechin gallate (EGCG) to prevent cognitive decline in *APOE*‐ ɛ4 carriers with Subjective Cognitive Decline: a randomized, double‐blinded clinical trial

**DOI:** 10.1002/alz.088694

**Published:** 2025-01-09

**Authors:** Laura Forcano, Natalia Soldevila‐Domenech, Anna Boronat, Thais Lorenzo, Aida Cuenca‐Royo, Nieves Pizarro, Albert Puig‐Pijoan, Iris Piera, Maria Gomis, Patricia Diaz‐Pellicer, Ana Aldea, Oriol Grau‐Rivera, Gonzalo Sánchez‐Benavides, Karine Fauria, Carolina Minguillon, Juan Domingo Gispert, Jose Luis Molinuevo, Rafael de la Torre

**Affiliations:** ^1^ Integrative Pharmacology and Systems Neurosciences Research Group, Neurosciences Research Program, Hospital del Mar Research Institute, Barcelona Spain; ^2^ Integrative Pharmacology and Systems Neurosciences Research Group, Neurosciences Research Program, Hospital del Mar Medical Research Institute (IMIM), Barcelona Spain; ^3^ Department of Medicine and Life Sciences. Pompeu Fabra University, Barcelona, Barcelona Spain; ^4^ Barcelona Beta Brain Research Center, Barcelona, Barcelona Spain; ^5^ Servei de Neurologia, Hospital del Mar, Barcelona Spain; ^6^ Integrative Pharmacology and Systems Neuroscience. Neuroscience Program. Hospital del Mar Medical Research Institute, Spain, Barcelona Spain; ^7^ Clinical Research Unit. Hospital del Mar Research Institute, Barcelona, Barcelona Spain; ^8^ Barcelonaβeta Brain Research Center (BBRC), Barcelona Spain; ^9^ Centro de Investigación Biomédica en Red de Fragilidad y Envejecimiento Saludable (CIBERFES), Instituto de Salud Carlos III, Madrid Spain; ^10^ Barcelonaβeta Brain Research Center, Barcelona, Spain, Barcelona Spain; ^11^ Hospital del Mar Research Institute (IMIM), Barcelona Spain; ^12^ Centro de Investigación Biomédica en Red de Fragilidad y Envejecimiento Saludable (CIBERFES), Madrid Spain; ^13^ Barcelonaβeta Brain Research Center (BBRC), Pasqual Maragall Foundation, Barcelona Spain; ^14^ Hospital del Mar Medical Research Institute (IMIM), Barcelona Spain; ^15^ Centro de Investigación Biomédica en Red de Fragilidad y Envejecimiento Saludable (CIBERFES), 28089, Madrid Spain; ^16^ Centro de Investigación Biomédica en Red Bioingeniería, Biomateriales y Nanomedicina (CIBER‐BBN), Instituto de Salud Carlos III, Madrid Spain; ^17^ Hospital del Mar Research Institute, Barcelona, Barcelona Spain; ^18^ Lundbeck A/S, Copenhagen Denmark; ^19^ Department of Medicine and Life Sciences, University Pompeu Fabra, Barcelona Spain; ^20^ Centro de Investigación Biomédica en Red Fisiopatología de la Obesidad y la Nutrición (CIBEROBN), Madrid Spain

## Abstract

**Background:**

Lifestyle interventions targeting multiple Alzheimer’s disease (AD) risk factors are effective strategies to prevent cognitive decline. Emerging evidence suggests synergistic effects between various intervention components, including lifestyle modifications, supplements, and pharmacological approaches. The PENSA study, part of the World‐Wide FINGERs network, is a randomized, double‐blind clinical trial following the Finger 2.0 Model. This work aims to show the results of the effectiveness of this 12‐month intensive personalized multimodal lifestyle intervention (MI) encompassing diet, physical activity, cognitive/social stimulation combined with epigallocatechin gallate (EGCG), in mitigating cognitive decline in individuals with Subjective Cognitive Decline (SCD) carriers of the APOE‐e4 allele. Aspects related to the nature and intensity of the intervention that might influence cognitive improvements will be discussed.

**Method:**

129 individuals aged 60‐80 years, APOE‐ε4 carriers meeting SCD, were enrolled and randomized into MI+EGCG (N = 52) or MI+placebo (N = 52), or allocated to a control group (CG) (receiving healthy lifestyle recommendations) (N = 25). The primary efficacy outcome was cognitive performance measured with the modified PACC‐exe battery. eHealth and mHealth tools/devices were used for the continuous monitoring of MI.

**Result:**

Although specific numerical results are not reported due to the upcoming submission for peer‐reviewed publication, we can anticipate that the results reflect promising results in cognitive performance, among participants undergoing MI + EGCG and a reduction in the risk of dementia as measured by the LIBRA index. Adherence to intervention components was very high (90.3% nutrition, 62.1% gymnasium, 72.6% cognitive training, 79.0% psychoeducation sessions, 89.0% daily ecological momentary assessments). Global findings will be shared in their integrity during the meeting.

**Conclusion:**

The PENSA Study is the first clinical trial exploring the effectiveness of integrating an intensive personalized MI with a pro‐cognitive complement, EGCG, in slowing cognitive decline in APOE‐ɛ4 carriers with SCD. Our findings emphasize the feasibility and effectiveness of a personalized MI for cognitively healthy individuals at higher risk of AD, applying an intense approach and incorporating new technologies. In the upcoming discussion, we will delve into the results and their broader implications.